# Spatial and temporal dynamics of virus occurrence in two freshwater lakes captured through metagenomic analysis

**DOI:** 10.3389/fmicb.2015.00960

**Published:** 2015-09-15

**Authors:** Mohammad Mohiuddin, Herb E. Schellhorn

**Affiliations:** Department of Biology, McMaster UniversityHamilton, ON, Canada

**Keywords:** metagenomics, virus like particles (VLPs), environmental DNA (eDNA)

## Abstract

Viruses are the most abundant microorganisms in the aquatic environment, yet the identification of viruses and assessing their diversity still remains a challenge. Here, we present a robust, routinely usable approach to identify viruses from two freshwater lakes of the lower Great Lakes region, Lake Ontario, and Lake Erie. We collected water samples from six different beaches of these two lakes during the summer period of 2012 and 2013, and separated into three distinct fractions, namely a bacterial fraction, a virus like particle (VLP) fraction, and a fraction of eDNA (environmental DNA). DNA extracted from all three fractions was sequenced and bioinformatic analyses of sequences revealed the presence of viruses from major viral families. The analyzed viral sequences were dominated by bacteriophage sequences, but also contained many plant and animal viruses. Within the context of this study, geographic location does not appear to have a major impact on viral abundance and diversity, since virome composition of both lakes were similar. Comparative analyses between eDNA and viral fractions showed that eDNA can be used in combination with VLP fractions to identify viruses from the environment.

## Introduction

Viruses, including phages outnumber microbial cells ten to one in most aquatic environments (Chibani-Chennoufi et al., 2004). At a concentration of 10^6^ to 10^9^ viruses per milliliter of sea water (Bergh et al., 1989) and up to 10^6^ viral species per kg of marine sediments (Breitbart et al., 2004) and 10^9^ viruses per gram of soil in terrestrial environments (Williamson et al., 2003), most likely, viruses are the largest reservoirs of underexplored microbial components of the entire biosphere. These large and diverse viral communities influence a number of processes ranging from global geochemical and ecological cycles (Fuhrman, 1999; Suttle, 2007) to bacterial virulence and pathogenesis (Brüssow et al., 2004). However, compared to marine viruses, nature of freshwater viral communities and their impact on aquatic environment still remains largely unexplored (Middelboe et al., 2008). Despite being underexplored, recent studies on the freshwater environment showing that freshwater viral communities are rich, consisting of diverse and novel viruses (Djikeng et al., 2009; Hewson et al., 2012; Roux et al., 2012).

The majority of viruses identified in freshwater environments is bacteriophages along with diverse human and animal viruses (Tseng et al., 2013). To a lesser extent, plant viruses, have also been detected in freshwater environments (Djikeng et al., 2009; Fancello et al., 2013; Tseng et al., 2013). Identification of these viruses, however, is challenging, as most of them are difficult to culture through traditional techniques. Unlike, 16S rRNA sequences of prokaryotes and 18S rRNA sequences of eukaryotes, the signature sequences shared by cellular organisms, viruses lack universally conserved sequences (Rohwer and Edwards, 2002). The lack of a universal phylogenetic marker limits direct detection of viruses in the environment. To address these limitations, viral fractions were isolated from freshwater and analyzed using shotgun sequencing and metagenomic analysis. Sequencing of whole viral communities has led to the rise of viral metagenomics (Breitbart et al., 2002) and provides an unprecedented insight into viral diversity. This culture-independent approach also overcomes many limitations of the culture based and/or PCR based methods for virus identification and offers an unrestricted access to the type and distribution of viruses in any host environment. More recently, because of the rapid development and cost reduction of sequencing techniques, metagenomic studies are being used regularly to identify viruses from the environment. However, few studies about viral content have been performed in the lower Great Lakes region, particularly in Lake Ontario and Lake Erie. Culture based and PCR based approaches used in these lakes have provided evidence for the presence of pathogenic microorganisms (Edge and Hill, 2007; Fong et al., 2007; Khan et al., 2014). Nevertheless, little is known about the type of viruses with the exception of algal viruses (Short et al., 2011) and coliphages (Fong et al., 2007). These lakes are of long standing interest as they are frequently used for recreational activities during the summer period and also a primary source of drinking water across the lower Great Lakes region. The study of viruses through metagenomic approach in this region, may provide an important insight into virus type and distribution.

Metagenomic studies applied to the viral populations of discrete environments have expanded our knowledge of viral diversity in recent years. However, comparison between diverse viromes is difficult because of the lack of a single methodology that allows the targeting of an entire viral population in a given environment. Diversity in viral nucleic acid composition (DNA or RNA, single-stranded or double-stranded) also limits their simultaneous detection. Traditionally, virome studies focus on either DNA or RNA viruses, where particles that pass through the 0.2 μm pore-size filters (Thurber et al., 2009) are considered as viruses. Filtration is an important step as it excludes most cellular fractions from viruses but it also leads to the exclusion or under-representation of giant viruses with diameter larger than 200 nm (Fischer et al., 2010; Arslan et al., 2011; Philippe et al., 2013). Therefore, it is important to employ a systematic approach which allows targeting all viruses within a given sample. So far, viral metagenomic studies did not consider the fact that viruses release free nucleic acids in the environment after lysis (natural decay or lysis) and extracellular DNA can persist in the environment for up to 1 month (Dejean et al., 2011). eDNA has been used to identify non-viral organisms from the environment (Ficetola et al., 2008; Thomsen et al., 2012) and therefore has the potential to provide valuable information about all viruses that may present in an environment. Here, we present a simple and systematic approach for the identification of viruses from freshwater sample using VLP DNA and eDNA. To identify and assess the diversity of the lower Great Lakes virome content, we collected water samples from six different beaches of Lake Ontario and Lake Erie from June to September of the year 2012 and 2013. These beaches are of special interest because they are heavily used for recreational activities during the summer period and are subject to frequent closures due to high *E. coli* counts. The virome content of Lake Ontario was further compared to that of Lake Erie to determine the effect of geographical location on the virome content.

## Materials and methods

### Sampling sites and sample collection

Water samples were collected in sterile 1.0 L sampling bottles (Nalgene) from six different locations across Lake Ontario and Lake Erie. Samples were collected from the surface at 1.0 m depth from Lakeside Beach, Queen's Royal Beach and Fifty Point Beach of Lake Ontario and from Long Beach, Long Beach Conservation Area East and Nickel Beach of Lake Erie during the summer period of 2012 and 2013 (Figure S1).

### Sample fractionation

**Figure 1:**
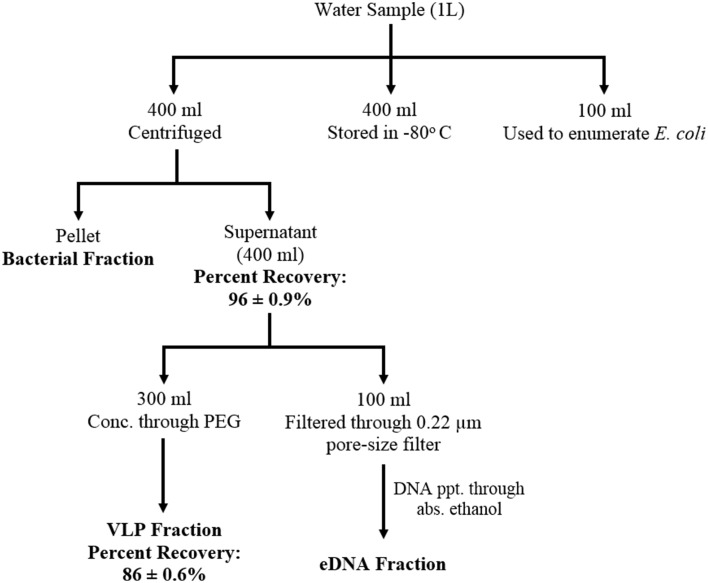
**Schematic diagram showing water sample fractionation steps and the efficiency of the fractionation scheme**. Water samples were separated into three distinct fractions: bacterial, VLP (Virus like particles), and eDNA fraction. eDNA is defined as the DNA released in the environment after lysis (natural decay or lysis) of bacteria, viruses, and higher organisms The efficiency of the fractionation scheme was determined as percent recovery of bacteriophage (MHS 16) mixed with the water sample prior to the fractionation steps.

After collection, samples were kept in ice and transferred to the lab within 4 h and processed within 24 h. Samples were separated into three distinct fractions: bacterial fraction, VLP (Virus like particles) fraction and eDNA fraction according to the schematic diagram (Figure 1). eDNA is defined as the DNA released in the environment after lysis (natural decay or lysis) of bacteria, viruses, and higher organisms. Briefly, 400 ml of water samples were centrifuged for 10 min at 10,000 × g at 4°C. After centrifugation, each pellet was resuspended with 2.0 ml of 1x PBS buffer (pH 7.2) and stored in −20°C as bacterial fraction. Of the 400 ml supernatant, 300 ml was used to concentrate the VLPs using PEG (poly-ethylene glycol) and MgSO_4_ at a final concentration of 4% and 15 mM respectively, (Colombet et al., 2007; Branston et al., 2012). The supernatant mixed with PEG and MgSO_4_was incubated at 4°C for 16 h and the mixture was then centrifuged for 30 min at 10,000 × g at 4°C to concentrate the VLPs. After concentrating the VLPs, the pellet (VLPs) was resuspended with 8.0 ml of 1x PBS (pH 7.2). The mixture was then filtered through low-protein-binding 0.22-μm-pore-size filter (Millex-GP; Millipore, Etobicoke, ON) and centrifuged at 180,000 × g for 1.5 h at 4°C. After centrifugation, the pellet was resuspended with 300 μl 1x PBS (pH 7.2) buffer and stored in −80°C as VLP fraction.

To isolate eDNA, 100 ml of the previously separated supernatant was filtered through 0.22 μm filter. The filtered supernatant was then mixed with a double volume of absolute ethanol and 1/10 th volume of 3M sodium-acetate (pH 5.2) and incubated at −20°C for overnight (Green et al., 2012). The mixture was then centrifuged (11,000 × g, 30 min; 4°C) and the supernatant was discarded. The pellet was resuspended with the remaining fluid (2–3 ml) inside the centrifuge bottle. The resuspended mixture was then incubated at −20°C for overnight and centrifuged (11,000 × g, 30 min; 4°C) again. After centrifugation, the supernatant was discarded and the pellet was washed with 1 ml of 70% ethanol (10,000 × g, 10 min; 4°C). The pellet was then dried and dissolved in 20 μl of 1x TE (pH 8.0) buffer and stored at −20°C as eDNA fraction.

### Extraction of DNA from bacterial and VLP fraction

Before extraction of the nucleic acids from VLP fraction, the supernatant was treated with DNaseI to remove extracellular DNA as PEG is known to precipitate extracellular DNA (Paithankar and Prasad, 1991). The DNase I treated supernatant was then used to extract DNA using Genomic DNA Isolation Kit (Norgen Biotek, Thorold, ON). The isolated DNA was further concentrated using a double volume of absolute ethanol and 1/10th volume of 3M sodium-acetate (pH 5.2) as described earlier and dissolved in 20 μl of 1x TE (pH 8.0). The concentrated DNA was then stored at −20°C. The concentration of the DNA samples was measured using Qubit 2.0 Fluorometer (Invitrogen). DNA from bacterial fractions was isolated using Soil DNA Isolation Kit (Norgen Biotek, Thorold, ON) according to the manufacturers' protocol.

### Preparation of DNA samples for sequencing

After extraction, DNA samples were diluted to a concentration of 0.2 ng/μl and libraries were prepared using Nextera XT DNA Sample Prep Kit (Illumina). Fragment size distribution of each library was checked using Bioanalyzer and High Sensitivity DNA Kit (Agilent). Samples were then normalized according to the supplier's instruction using Nextera XT DNA Sample Prep Kit. Five microliters of each library was pooled and concentrated. The final pool of each library was then quantified using qPCR and sequenced using 100 bp paired-end, HiSeq 2000, Illumina platform located at Farncombe Institute (McMaster University, Hamilton, ON, Canada). Sequence statistics listing number of libraries used and number of reads generated are included as supplementary information (Datasheet S1). Sequences have been submitted to the NCBI SRA database under the Study Accession SRP060006, Bioproject Accession PRJNA288501 and the accession number of each library has been included as supplementary information (Datasheet S1).

### Bioinformatic analyses

Paired-end sequence reads generated from the Illumina HiSeq were assembled into contigs using CLC Genomics Workbench version 6.5.2 (CLC bio, Boston, MA, USA). Assembly was performed using *de novo* assembly with automatic word and bubble sizes, a minimum of 66 nucleotides in the reads, mismatch cost set to 2, length fraction set to 0.5, and similarity fraction set to 0.8. In addition, colorspace error cost, insertion and deletion cost were set to three for assembly. Contigs with a length of less than 200 bp were not considered for analysis. The contigs were then submitted to the Metavir (http://metavir-meb.univ-bpclermont.fr/) pipeline for phylogenetic analysis (Roux et al., 2014). Raw sequences were also uploaded and analyzed using MG-RAST server (http://metagenomics.anl.gov/), an online metagenome annotation pipeline (Meyer et al., 2008). Before uploading, sequences were quality trimmed using MG-RAST QC pipeline, which includes removal of artificial or technical replicates (Gomez-Alvarez et al., 2009) and removal of low quality sequences (Cox et al., 2010). Gene-calling was performed using automated pipeline in MG-RAST which includes the use of FragGeneScan (Rho et al., 2010), clustering of predicted proteins at 90% identity by using uclust (Edgar, 2010) and the use of sBLAT, an implementation of the BLAT algorithm (Kent, 2002) for similarity analysis of each cluster. Sequence reads for which gene prediction tools could not identify a match, are considered as “unknown” which is divided into two categories—unknown sequences (sequences with no similarity to any protein or rRNA sequence) and unknown proteins (predicted proteins of unknown functions, Meyer et al., 2008). Taxonomic composition of the viromes were obtained through comparison of sequences to the curated NCBI RefSeq complete viral genomes protein sequence database using blastX with an *e*-value cut-off of ≤ 10^−3^. Taxonomic composition was deduced from the best blast hit of each contig.

## Results

### Efficiency of the fractionation scheme

Efficiency of the fractionation scheme was determined using spiking experiment. A previously isolated bacteriophage MHS-16 (Host Strain: *E. coli* K-12) was spiked into the test water sample (which tested negative for the presence of any *E. coli* K-12 bacteriophage) and percent recovery of the phage was determined through enumeration of phage at every fractionation step (Figure 1). Briefly, MHS-16 was added to 400 ml water sample at a titer of 7.5 × 10^6^ PFU/ml. Five hundred microliters of each fractionated sample (supernatant or resuspended pellet, filtered through 0.22 μm-pore-size filter) was then serially diluted (10-fold dilution) and used to enumerate bacteriophage using double agar overlay method (Green et al., 2012). One hundred microliters of the diluted phage particle was mixed with 500 μl of the logarithmic-phase host cells and 3.5 ml aliquots of 0.4% soft agar. The mixture was then poured on to the nutrient agar plate (LB agar) and incubated overnight. Recovery of phage, calculated by counting the number of plaques formed, was 86 ± 0.6% of the inoculated bacteriophage from spiked VLP fractions. The recovery was slightly low when compared with the recovery from purified phage suspension (~ 95%, data not shown) and this decrease may be due to the presence of inhibitory agents (such as organic molecules, clay minerals or charged particles) in the environment.

### DNA in the environmental water samples

**Figure 2:**
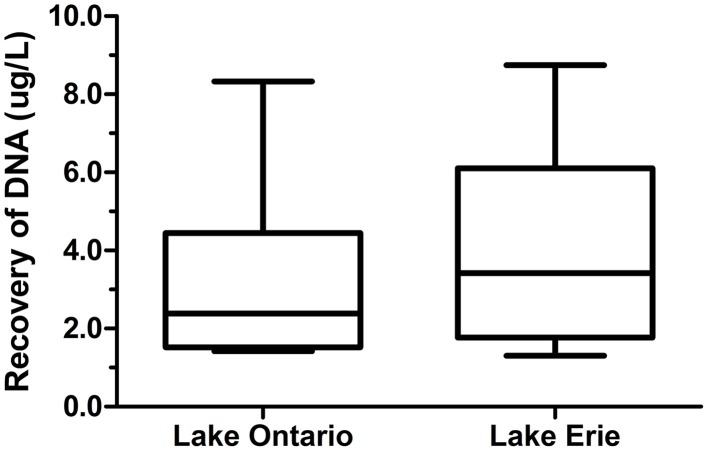
**Recovery of DNA from freshwater environment**. DNA was extracted from three different fractions of 12 individual 1 L samples. Six samples from Lake Ontario (four samples from Lakeside Beach, one sample from Queen's Royal Beach and Fifty Point Beach) and six samples from Lake Erie (four samples from Long Beach and one sample from Long Beach Conservation Area East and Nickel Beach) were used to extract DNA and amount of DNA recovered from all three fractions were normalized to 1 L water sample.

**Figure 3:**
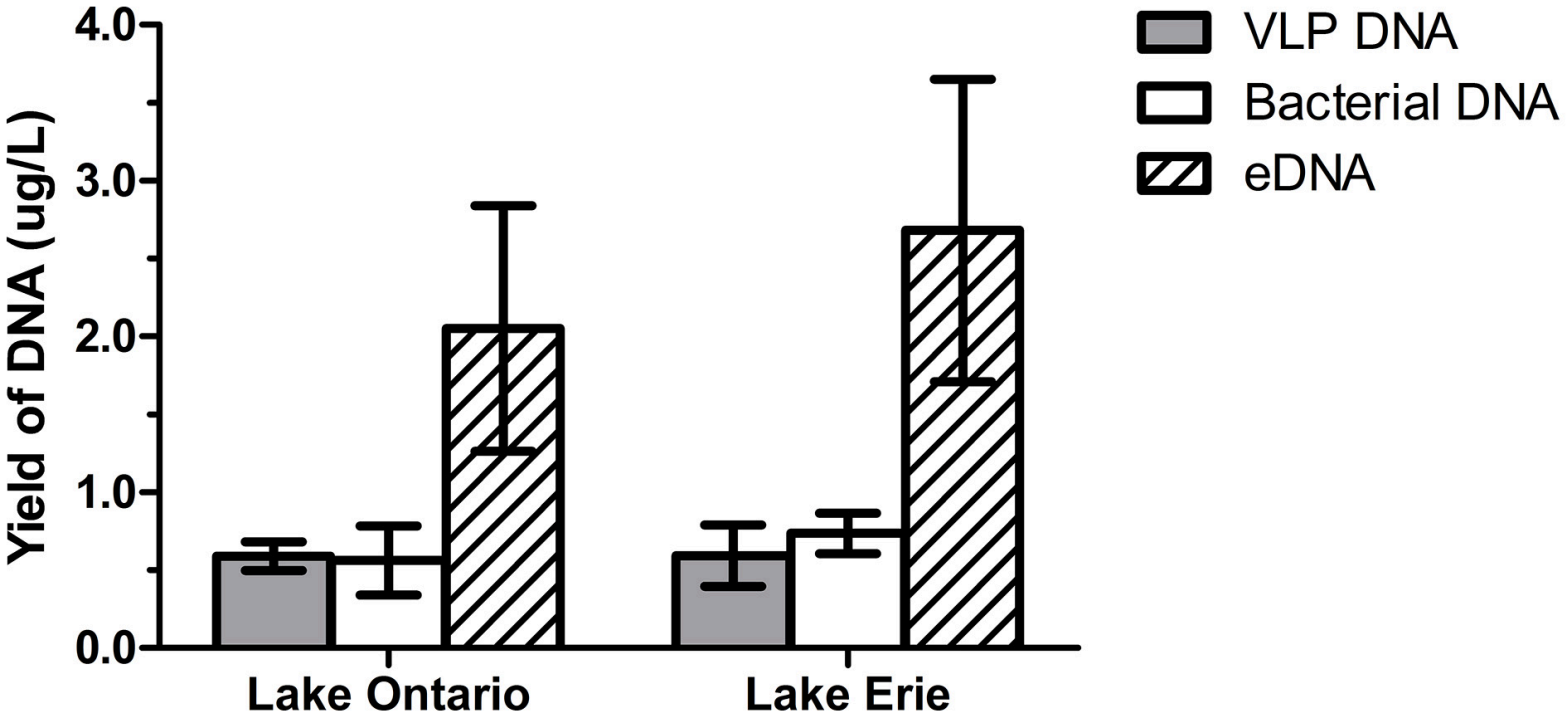
**DNA yield from VLP, bacterial, and eDNA fraction**. DNA recovered from six samples from Lake Ontario (four samples from Lakeside Beach, one sample from Queen's Royal Beach and Fifty Point Beach) and six samples from Lake Erie (four samples from Long Beach and one sample from Long Beach Conservation Area East and Nickel Beach) were used to calculate DNA yield from each fraction. eDNA accounts for most of the DNA (~60%) in freshwater environment while VLP DNA and bacterial DNA are present in almost equal amount.

The amount of DNA recovered from isolated fractions was determined from the collected water samples of Lake Ontario and Lake Erie. A total of 12 samples (six samples from each lake) was used to extract DNA from all three fractions. Recovery of DNA varied from 1.0 to 9.0 μg DNA from 1 L water samples (Figure 2 and Datasheet S2). We also estimated the total DNA of each fraction and found that eDNA accounts for more than half (58.5 ± 5.1%) of the total DNA in a given sample, whereas bacterial and VLP fractions account for 20 ± 3.6% and 21.5 ± 4.8% respectively, (Figure 3).

### Abundance of bacterial and viral genome copies in freshwater

Number of bacterial and viral genome copies in freshwater environment was calculated by summing the DNA content from both bacterial and VLP fractions. Six samples from Lake Ontario (four samples from Lakeside Beach, one sample from Queen's Royal Beach and Fifty Point Beach) and six samples from Lake Erie (four samples from Long Beach and one sample from Long Beach Conservation Area East and Nickel Beach) were used to estimate the DNA amount. Assuming that average viral genome size of 50 kb length (dsDNA) (Steward et al., 2000), we converted the DNA mass to genome copy equivalent and found that the number of viral genome copies vary from 7 to 15 × 10^6^ per milliliter of water sample which is consistent with previously published viral metagenomes (Bergh et al., 1989; Yoshida et al., 2013) (Table 1). We also estimated the number of bacterial genome copies assuming the average bacterial genome size of 4.7 Mb (Raes et al., 2007; Angly et al., 2009) and found that number of bacterial genomes varies from 1.8 × 10^4^ to 1.1 × 10^5^ per milliliter of water sample (Table 1).

**Table 1:** **Relative abundance of bacteria and viruses in freshwater**.

**Average genome copies**	**Lake Ontario**	**Lake Erie**
Viral genome copies (× 10^6^ ml^−1^)	10 (±2.7)	9 (±6.5)
Bacterial genome copies (× 10^4^ ml^−1^)	3 (±1.2)	8.9 (±2.0)

### Overview of the viromes

**Figure 4:**
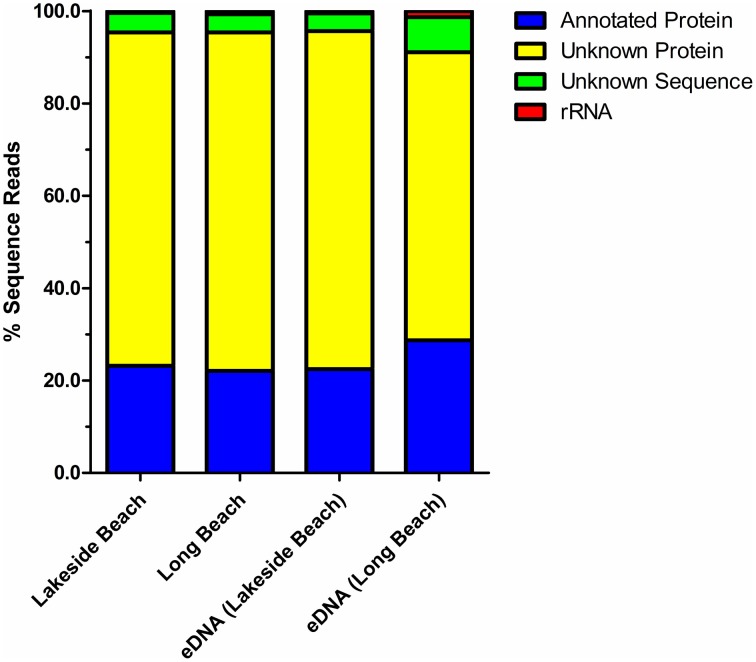
**Annotation of the virome sequence reads**. Sequences from both VLP DNA and eDNA fractions of Lakeside and Long Beach samples (collected in 2012) were compared against the non-redundant M5NR database (*E*-value < 10^−3^ in blastX). About 25% of the sequence reads mapped to the known protein sequences of M5NR database while majority of the sequence reads were categorized as unknown. Among the unknown reads, unknown sequences are sequences for which gene prediction tools could not predict any protein or rRNA sequence and unknown proteins represent predicted proteins with unknown functions.

**Figure 5:**
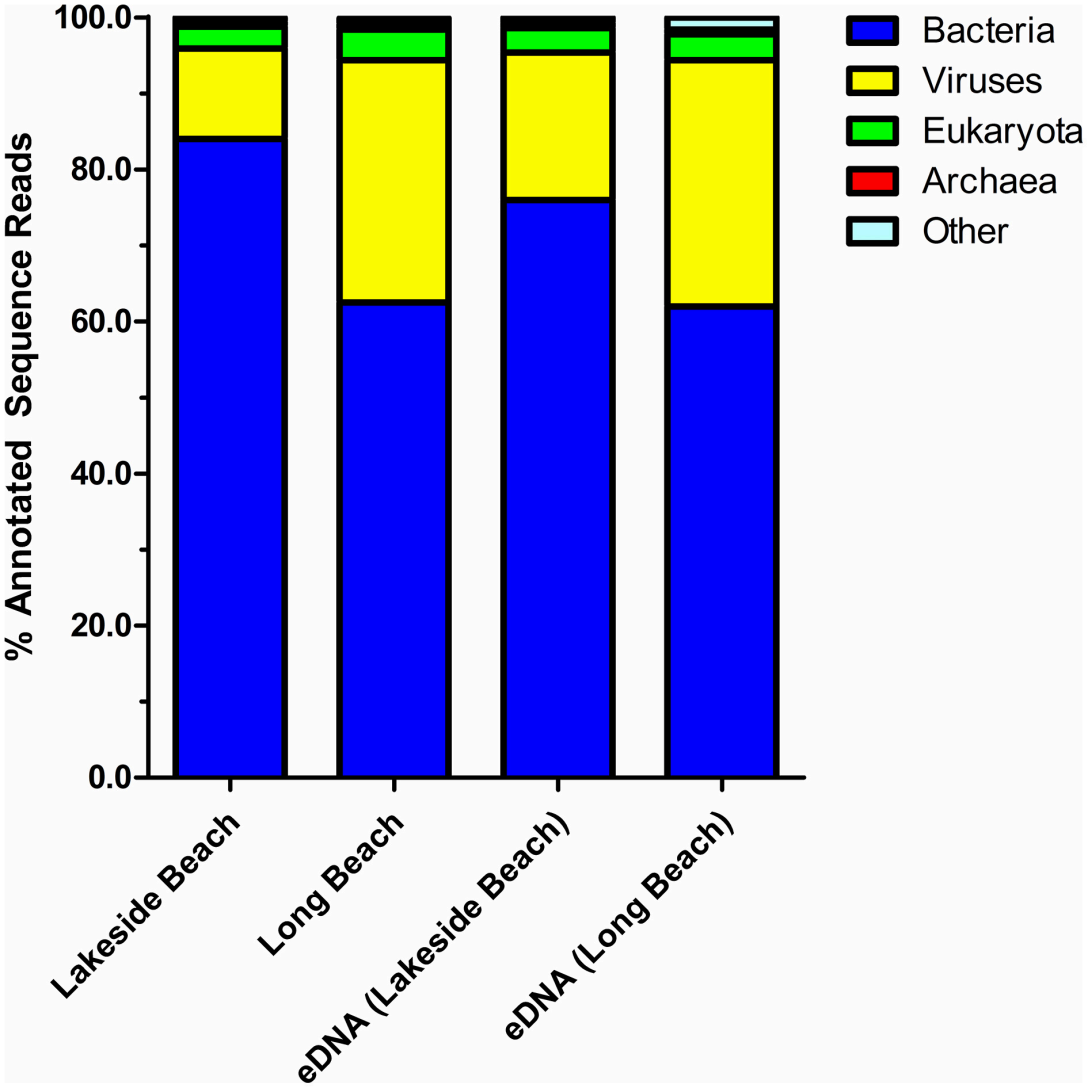
**Taxonomy of virome domains**. Sequence reads from Lakeside Beach and Long Beach VLP DNA and eDNA fractions (of the year 2012) were compared against the M5NR database (*E*-value < 10^−3^ in blastX). Majority of the sequence reads (60–80%) mapped to bacterial sequences and 20–35% sequence reads mapped to virus sequences of the database.

Percentage of sequences that show homology to the sequences in public databases was determined by uploading the sequence reads to MG-RAST and compared against the non-redundant M5NR database (Wilke et al., 2012). Consistent with the previously published freshwater viral metagenomes (Hewson et al., 2012; Roux et al., 2012), majority of our sequences did not show any homology to the known protein databases (ORFans, Figure 4). The percentage of ORFans was slightly higher in Lakeside Beach viromes than the Long Beach viromes. Sequences with similarity to the known databases are known as ORFs and ORFs from the viral metagenomes were further divided into five major domains, namely, bacteria, viruses, eukaryotes, archaea and other. “Other” refers to sequences originating from mobile genetic elements such as plasmids and cloning vectors. Bacterial sequences predominated the viromes from both Lakeside and Long Beach samples (84% in Lakeside and 62% in Long Beach) (Figure 5). Viruses accounted for approximately 32% in Long Beach, and 12% in Lakeside Beach samples.

### Taxonomic composition of the viromes

**Figure 6:**
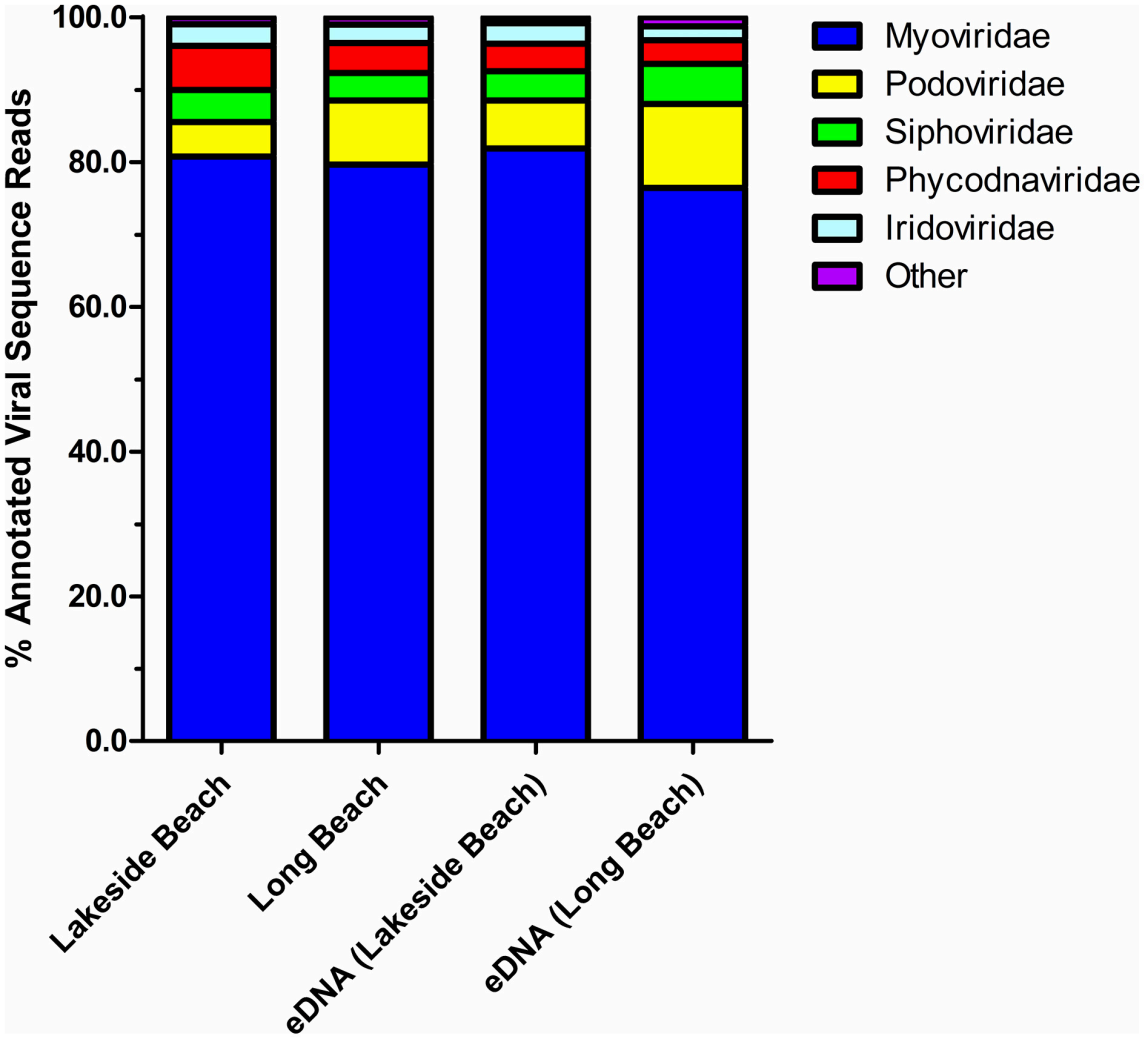
**Relative abundance of top five viral families in the lower Great Lakes water samples**. blastX comparison (*E*-value < 10^−3^) of VLP DNA and eDNA sequences of Lakeside Beach and Long Beach samples (sampled in 2012). Unclassified viruses and viral families constituting less than 1% of total viral reads are categorized as “Other.”

Taxonomic composition of the viral communities was determined through comparison of sequence reads against the curated non-redundant RefSeq Virus database using blastX with an *e*-value threshold of ≤ 10^−3^. Among annotated sequences, viruses of the *Myoviridae* family comprised the greatest proportion (79.7 ± 1.2%) whereas *Podoviridae* and *Siphoviridae* family comprised the second and third largest proportion of sequences, respectively (7.9 ± 1.5% and 4.5 ± 0.4% respectively, Figure 6, Tables S1, S2). Viruses belong to these three families are dsDNA phages that infect only bacteria and therefore, majority of the viruses (~ 90%) in both Lakeside and Long Beach viromes are bacteriophages. However, algal viruses (*Phycodnaviridae*) and insect (or animal) viruses of the *Iridoviridae* family were also present (4.3 ± 0.6% and 2.6 ± 0.2%, respectively). Apart from these major viral families, viruses belonging to other families such as *Asfarviridae, Poxviridae, Herpesviridae, Mimiviridae*, and ssDNA phages of *Microviridae* and *Inoviridae* family are also present in these viromes (Tables S1, S2).

### Comparison of the viromes

To determine the impact of geographical location on the distribution of viruses, we compared the virome content between six different beaches of Lake Ontario and Lake Erie. We did not observe any major difference in virome content among the six beaches tested suggesting that geographical location may not be a major determinant (within the context of this study) on the diversity of the viruses in the lower Great Lakes region. However, additional sampling is required to further validate this finding. Viruses belonging to the *Myoviridae* family were predominant (~ 80%) in all beach sites whereas viruses of the *Podoviridae, Siphoviridae, Phycodnaviridae*, and *Iridoviridae* family comprised the majority of the rest viral sequences (Table S2). Relative abundance of viral families in eDNA fractions were consistent with the Lake Ontario and Lake Erie viromes (Table S1) except viruses of the *Podoviridae* family which is found in slightly higher concentrations in eDNA fractions.

The distribution of viruses in aquatic environment changes over time (Hewson et al., 2012) and extreme climatic conditions such as typhoons, heavy rainfalls can also change the composition of microbial communities (Tseng et al., 2013). Change in host microbial community likely leads to commensurate changes in the viral community. Therefore, to investigate the impact of the environmental change and seasonal variation on the virome composition of the lower Great Lakes region, we compared the viromes of Lake Ontario and Lake Erie over the year 2012 and 2013. Viruses of the *Myoviridae* family, which still comprises the majority of the viruses in these lakes, their abundance dropped by 10–13% from 2012 to 2013 (Table 2) and viruses of the *Podoviridae* and *Siphoviridae* family increased. The relative abundance of other families remained similar over this period.

**Table 2:** **Temporal change of major viral families in the Lakeside and Long Beach viromes (VLP fraction)**.

**Virus Family**	**Primary Host**	**Relative abundance (% of viral reads)**			
		**Lakeside Beach**		**Long Beach**	
		**2012**	**2013**	**2012**	**2013**
*Myoviridae*	Bacteria	80.76	70.70	79.66	66.82
*Podoviridae*	Bacteria	4.74	11.88	8.81	14.25
*Siphoviridae*	Bacteria	4.41	6.13	3.83	7.71
Unclassified (*Caudovirales* order)	Bacteria	0.78	0.84	0.88	0.93
*Inoviridae*	Bacteria	–	–	0.01	–
*Phycodnaviridae*	Algae	6.12	4.83	4.12	4.44
*Iridoviridae*	Insects, Amphibians, Fish, Invertebrates	2.97	5.50	2.57	4.44
*Poxviridae*	Humans and other vertebrates, Arthropods	0.05	–	0.04	0.23
*Alloherpesviridae*	Fish, Aphibians	0.02	–	0.03	0.47
*Herpesviridae*	Animals, including humans	0.05	0.04	0.03	–
*Marseilleviridae*	Amoeba	0.06	0.04	0.01	0.23
*Baculoviridae*	Insects	0.03	0.04	0.02	0.23
*Adenoviridae*	Humans and other vertebrates	0.02	–	–	–
*Nimaviridae*	Crustaceans	0.02	–	–	0.23

To compare the diversity within each lake, biological replication was determined by testing DNA recovery and variation among the top five virus families identified in replicate samples taken at three sites within the same beach. Although the recovery of DNA from the fractions (VLP, bacterial, and eDNA fractions) varied highly among the replicates (Datasheet S2), this high variability did not affect the abundance of highly abundant viruses (Table 2, Tables S2, S3).

### Phylogenetic analysis of the viromes

**Figure 7:**
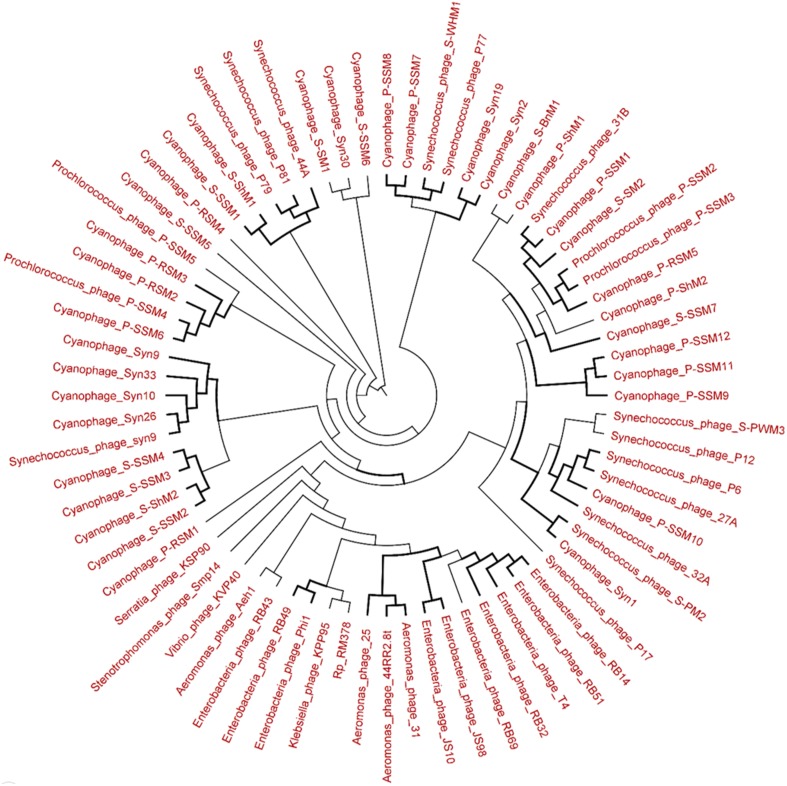
**Neighbor-joining phylogenetic tree of the capsid assembly protein G20 (T4-like phages or cyanophages) (pfam07230) of Lakeside Beach virome**. Lakeside-1 virome library was used as a representative metagenome profile for the Lakeside Beach site. Sequence reads with significant similarity (*E*-value < 10^−3^ using blastX) to the G20 marker sequences were obtained, assembled at 98% identity in 35 bp using Cap3 and used to draw the phylogenetic tree alongside reference sequences taken from the protein family (PFAM) database. Sequences for which the best blast hit did not correspond to the G20 marker were excluded from analysis. Bootstrap values of ≥80 are highlighted with black lines.

**Figure 8:**
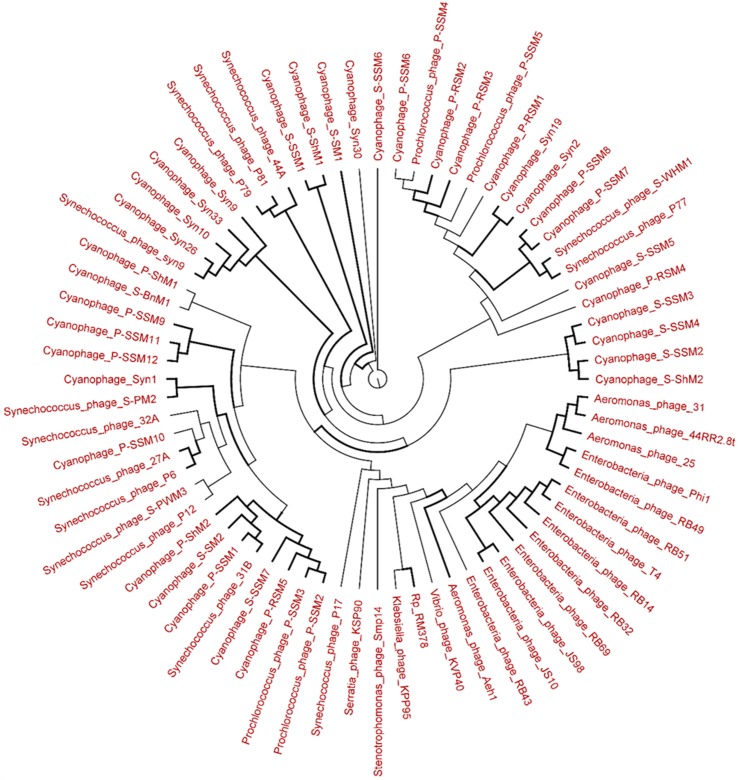
**Neighbor-joining tree of the capsid assembly protein G20 (T4-like phages or cyanophages) (pfam07230) of Long Beach virome**. Long Beach-1 virome library was used as a representative metagenome profile for the Long Beach site. Sequence homologs (*E*-value < 10^−3^ using blastX) to the G20 marker sequences were obtained from the virome library, assembled at 98% identity in 35 bp using Cap3 and used to draw phylogenetic tree alongside reference sequences taken from the protein family (PFAM) database. Sequences for which the best blast hit did not correspond to the G20 marker were excluded from analysis. Bootstrap values of ≥80 are highlighted with black lines.

**Figure 9:**
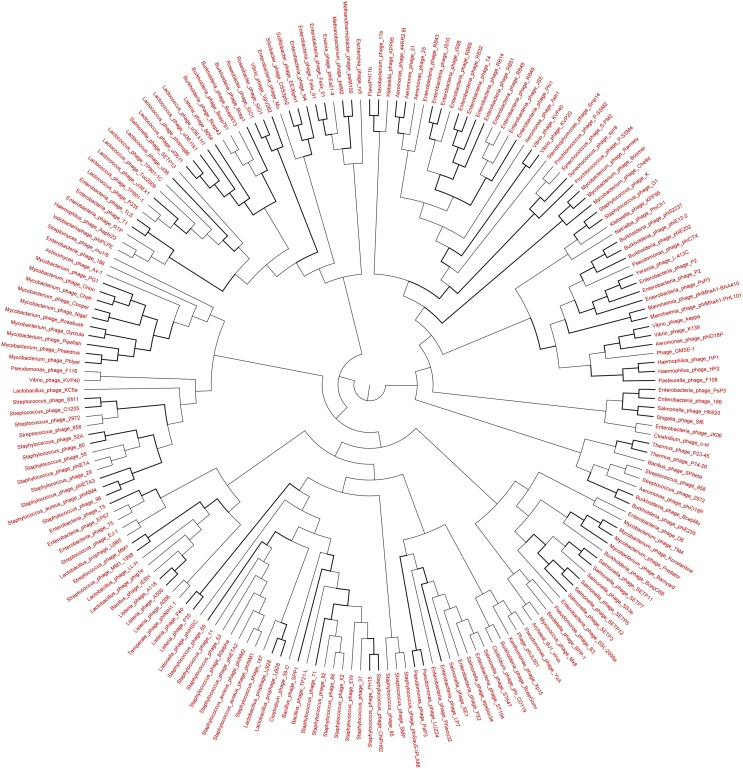
**Neighbor-joining phylogenetic tree of the TerL (Terminase Large subunit—for *Caudovirales* including *Myoviridae*, *Podoviridae*, and *Siphoviridae*) (pfam03237) of Lakeside Beach virome**. Lakeside-1 virome library was used as a representative metagenome profile for the Lakeside Beach site. Sequence reads with significant similarity (*E*-value < 10^−3^ using blastX) to the TerL marker sequences were obtained, assembled at 98% identity in 35 bp using Cap3 and used to draw phylogenetic tree alongside reference sequences taken from the protein family (PFAM) database. Bootstrap values of >80 are highlighted with black lines. Sequences for which the best blast hit did not correspond to the TerL marker were excluded from analysis. Bootstrap values of ≥80 are highlighted with black lines.

**Figure 10:**
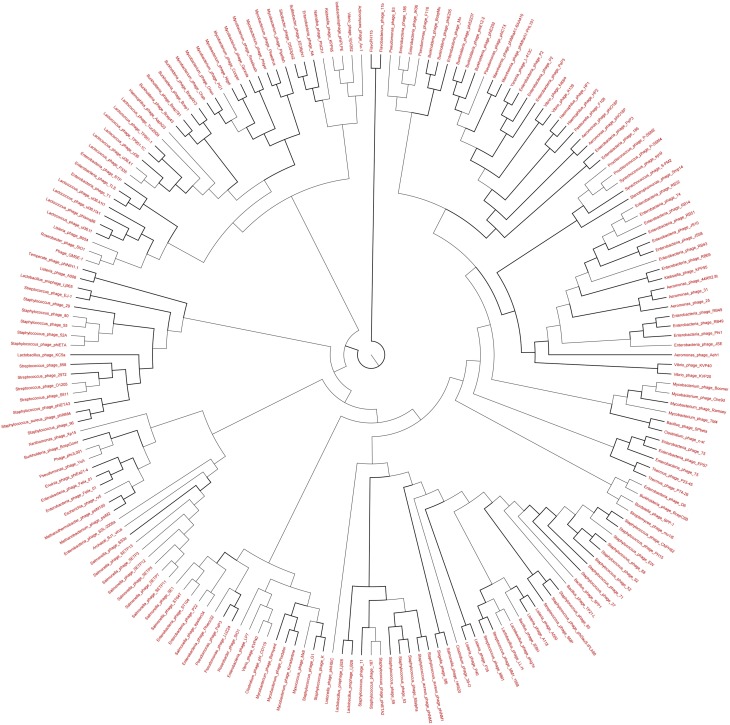
**Neighbor-joining phylogenetic tree of the TerL (Terminase Large subunit—for *Caudovirales* including *Myoviridae*, *Podoviridae*, and *Siphoviridae*) (pfam03237) of Long Beach virome**. Long Beach-1 virome library was used as a representative metagenome profile for the Long Beach site. Sequence homologs (*E*-value < 10^−3^ using blastX) to the TerL marker sequences were obtained from the virome library, assembled at 98% identity in 35 bp using Cap3 and used to draw phylogenetic tree alongside reference sequences taken from the protein family (PFAM) database. Sequences for which the best blast hit did not correspond to the TerL marker were excluded from analysis. Bootstrap values of ≥80 are highlighted with black lines.

The genetic diversity of the Lakeside Beach and Long Beach viromes (two representative beaches of Lake Ontario and Lake Erie, respectively) were examined with the automated Metavir tool. Based on the Metavir data, two types of phylogenetic markers were used: G20, a well-known marker to assess the diversity of T4-like phages (or cyanophages) (Dorigo et al., 2004; Wilhelm et al., 2006) and TerL to assess the diversity of *Caudovirales* including *Myoviridae, Siphoviridae*, and *Podoviridae* as these three families comprise over 90% of total viruses identified in the samples. Phylogenetic trees of the G20 and TerL sequences obtained from Lakeside and Long Beach viromes are shown in Figures 7–10. Phylogenetic tree analyses did not indicate any major difference between Lakeside and Long Beach viromes but minor differences were seen in branch length in these viromes (For example, branch length of Cyanophage-S-SM1, Syn30, and S-SSM6 differs in Lakeside and Long Beach viromes, Figures 7, 8). These differences may be due to the difference in nucleic acid composition of these viruses. However, as full length genome sequences were not used to construct these trees, it is very difficult to comment on the variation in genetic makeup of these viruses.

## Discussion

In this pilot survey study, we employed a metagenomic approach to explore the diversity of viruses from representative sites in the lower Great Lakes region. Only a few species of viruses (or phages) have previously been identified in samples from this area (Fong et al., 2007; Short et al., 2011). As viruses infect all living beings, vital players in global geochemical and ecological cycling of key nutrients (C and N, Suttle, 2005, 2007) and help maintaining balance in aquatic microbial communities (Rohwer and Thurber, 2009), exploring the nature of viral communities in the Great Lakes region may provide important insight into their role in shaping the microbial communities as well as in fixing and cycling of carbon and other nutrients. Here, we used a combinatorial approach using both VLP DNA and eDNA to fully capture the viral diversity. Resultant analyses of the viromes indicated high viral concentration (> 10^7^ virus like genome equivalent per ml) in water samples collected from six different beaches across Lake Ontario and Lake Erie. To our knowledge, this is the first such study that employs eDNA, in combination with the VLP DNA to examine freshwater viral communities. eDNA has been used to identify non-viral species from freshwater environments (Ficetola et al., 2008; Thomsen et al., 2012), but it has never been used in the identification of viruses from aquatic ecosystem and our analysis suggests that eDNA can represent the major viral groups in a given sample (Table S1). We determined the amount of DNA in the water sample and found that eDNA accounts for ~60% of all the DNA, whereas VLP and bacterial fractions account for ~21 and ~20%, respectively. Apart from bacterial and VLP DNA, eukaryotes such as plants and animals contribute to the high proportion of eDNA in water samples (Nielsen et al., 2007) and therefore, eDNA can be used to identify many organisms in the environmental samples.

Sample fractionation facilitates the separation and selective enrichment of viruses from the entire microbial community. We employed a fractionation scheme to identify all microorganisms, including viruses and bacteria from the environmental water samples. In contrast to other viral metagenomic studies, our fractionation scheme does not require large volumes of water samples (300 ml instead of 20–100 L, Thurber et al., 2009; Roux et al., 2012). To separate the VLP fraction, only 300 ml of water sample was concentrated and the virome obtained represents the majority of viruses that may be present in a given environment. The majority of the sequences (65–75%) from our viral metagenomes did not map to the publicly-available database (M5NR, Figure 4). Presence of unknown viruses (or bacterial cells), in aquatic environment results in high percentage of unknown sequences in viral metagenomes (Breitbart et al., 2002; López-Bueno et al., 2009). However, as sequence read length is also known to influence estimates of the abundance of unknown sequences (Wommack et al., 2008), shorter sequences (~ 100 bp) generated by shotgun sequencing, certainly have contributed to the high percentage of unknown sequences.

The majority of annotated sequences, mapped to bacterial genomes (62–84%). Several factors may be responsible for this high percentage of bacterial sequences in viral metagenomes (Angly et al., 2006) including the presence of unknown prophages in bacterial genomes, phages carrying host genes, relatively large size of bacterial genomes compared to viral genomes and the larger size of the microbial genome database than viral genome database statistically increasing the chance of matching bacterial sequences. The percentage of viral sequences among the annotated sequences was higher in Long Beach viromes (32%) than Lakeside Beach viromes (12%).

The majority of the viruses (90%) in Lake Ontario and Lake Erie viromes are bacteriophages and belong to the three major viral families (*Myoviridae, Siphoviridae*, and *Podoviridae*) of bacteriophages (Figure 6, Table 2, Tables S1–S3). The high percentage of viruses (or phages) belonging to the *Myoviridae* family (~80%) is mainly due to the abundance of cyanophages and viruses of the abundant SAR11 and SAR16 (Pelagibacter) myoviruses. Genome size of the viruses belonging to these three major viral families ranges from 30 to 170 kb. However, genome size of the viruses belonging to the eukaryotic large NCLDVs (nucleocytoplasmic large DNA viruses) ranges from 100 kb to ~2.5 Mb. The NCLDVs are dsDNA viruses of the eukaryotes and include viruses from six families, including *Poxviridae, Asfarviridae, Iridoviridae, Ascoviridae, Mimiviridae*, and *Phycodnaviridae*. Among these six viral families, viruses belonging to the *Phycodnaviridae* family are mostly algal viruses and viruses of the other five families are mainly animal (or insect) viruses. All these six viral families share some core genes and likely have originated from a putative common ancestor (Iyer et al., 2001, 2006). Apart from these large viruses, viruses of relatively small genome size belonging to the *Microviridae* and *Inoviridae* family were also found in the Lake Ontario and Lake Erie beach viromes. Viruses of these two families are ssDNA phages and their genome size ranges from 4 to 9 kb.

Relative abundance of viral genotype changes over time (Hewson et al., 2012) and this was reflected in our 2012 and 2013 virome datasets. Viruses belonging to the *Myoviridae* family dropped from ~80 to ~70% in 2013 whereas, viruses of the *Siphoviridae, Podoviridae*, and *Iridoviridae* family increased. Viruses belonging to *Myoviridae, Siphoviridae*, and *Podoviridae* families are mainly bacteriophages. Therefore, the temporal dynamics of viral genotypes in these regions indicate a change in the microbial community as viruses depend on their hosts to survive and replicate. Viruses of the *Iridoviridae* family mainly infect invertebrates such as insects. However, viruses of vertebrates such as fish, reptiles and amphibians also belong to this family. The change in the abundance of these viral families may be due to factors such as climate change and precipitation events (Hewson et al., 2012; Tseng et al., 2013).

Our fractionation scheme, though efficient at capturing the majority of the viruses, may exclude some important viruses. As the efficiency of recovery was 86 ± 0.6%, some viruses may be lost during the concentration step of the virome preparation (Figure 1). This loss may lead to an under-representation of low abundant viruses or rare viruses in our datasets. Although, giant viruses of eukaryotes are present in our prepared viromes, filtration of water samples through 0.22-μm-pore-size filter is known lead to the under-representation of large viruses (Fischer et al., 2010; Arslan et al., 2011; Philippe et al., 2013). Our fractionation scheme also does not consider the RNA viruses that are abundant in the aquatic environments (Djikeng et al., 2009; Culley et al., 2014) and is, therefore, unable to generate a comprehensive profile of all viruses present in a given sample. Furthermore, eDNA, which accounts for approximately 60% of the total DNA per liter of water sample, the minimum amount of eDNA that is required to identify less-common species from the environment be further investigated.

Although the majority of virus types was captured through our fractionation protocol, more sampling is necessary to generate a comprehensive profile of all virus types. As RNA viruses cannot be detected simultaneously with DNA viruses using currently available techniques of virus discovery, with the advancement of technology, in the future, we hope to identify all viruses from lower Great Lakes water samples. From the metagenome libraries used in this study, we have assembled 205 complete viral genomes (Datasheet S1). Detailed characterization of these draft viral genomes, including their distribution and phylogenetic analyses, will be the subject of future investigation. In addition to viruses, identification of microbial populations using amplicon sequencing will provide valuable information regarding virus-host interaction, horizontal gene transfer and the emergence of pathogenic strains in these lakes. Frequent monitoring of seasonal and temporal variation of virus abundance can also provide important information about the nature of change in microbial community over a long period. The DNA content of freshwater samples is *a priori* unknown and may be highly variable. As only a few ng of DNA is needed to generate an Illumina HiSeq library (Binga et al., 2008) and about 1.0–9.0 μg DNA can be recovered from 1.0 L of freshwater sample, in future, we aim to use relatively smaller volume of water sample to identify microbial and viral communities from the environment. Complementary to current water analysis techniques, using low sample volume will allow municipal authorities to assess the quality of freshwater environments especially in regions where regular monitoring is conducted.

In this study, we partnered with municipal water monitoring authorities to assess whether additional information obtained in metagenomic analyses, provides useful information for source tracking and/or estimation of public health risk (through the identification of sequences associated with pathogens). For large or heavily-used sites (e.g., popular beaches), multiple sampling locations (up to 5) are monitored with a given site with only one sample taken at each sampling location At the beginning of this study, we considered samples taken at these sub site locations to be replicates as there is systematic differences among them. However, it is clear from this study that DNA content can vary considerably among sub site samples and, in future, additional sampling, not currently included in municipal sampling programs, will have to be done to obtain good estimates of reproducibility.

### Conflict of interest statement

The authors declare that the research was conducted in the absence of any commercial or financial relationships that could be construed as a potential conflict of interest.
