# Supplementary material for: Spatial and temporal dynamics of virus occurrence in two freshwater lakes captured through metagenomic analysis
Source: Front Microbiol. 2015 Sep 15;6:960. doi: 10.3389/fmicb.2015.00960 (PMC4569853; doi:10.3389/fmicb.2015.00960)
Supplement: Supplementary file 1 [file Table1.DOCX]

**Table S1| Major viral families in the Lakeside Beach and Long Beach viromes**

| **Virus Family** | **Primary Host** | **Relative Abundance (% of viral reads)** | | | |
| --- | --- | --- | --- | --- | --- |
|  |  | **VLP (Lakeside Beach)** | **VLP (Long Beach)** | **eDNA (Lakeside Beach)** | **eDNA**  **(Long Beach)** |
| *Myoviridae* | Bacteria | 80.76 | 79.66 | 81.84 | 76.43 |
| *Podoviridae* | Bacteria | 4.74 | 8.81 | 6.67 | 11.57 |
| *Siphoviridae* | Bacteria | 4.41 | 3.83 | 4.03 | 5.54 |
| Unclassified (*Caudovirales* order) | Bacteria | 0.78 | 0.88 | 0.63 | 1.16 |
| *Inoviridae* | Bacteria | - | 0.01 | - | 0.01 |
| *Microviridae* | Bacteria | - | - | - | 0.01 |
| *Phycodnaviridae* | Algae | 6.12 | 4.12 | 3.79 | 3.28 |
| *Iridoviridae* | Insects, Amphibians, Fish, Invertebrates | 2.97 | 2.57 | 2.85 | 1.93 |
| *Poxviridae* | Humans and other vertebrates, Arthropods | 0.05 | 0.04 | 0.06 | 0.03 |
| *Alloherpesviridae* | Fish, Amphibians | 0.02 | 0.03 | 0.07 | 0.02 |
| *Herpesviridae* | Animals including humans | 0.05 | 0.03 | 0.01 | 0.01 |
| *Asfarviridae* | Pigs | - | - | - | 0.01 |
| *Marseilleviridae* | Amoeba | 0.06 | 0.01 | - | 0.01 |
| *Baculoviridae* | Insects | 0.03 | 0.02 | 0.06 | - |
| *Adenoviridae* | Humans and other vertebrates | 0.02 | - | - | - |
| *Nimaviridae* | Crustaceans | 0.02 | - | - | - |
