# Supplementary material for: Spatial and temporal dynamics of virus occurrence in two freshwater lakes captured through metagenomic analysis
Source: Front Microbiol. 2015 Sep 15;6:960. doi: 10.3389/fmicb.2015.00960 (PMC4569853; doi:10.3389/fmicb.2015.00960)
Supplement: Supplementary file 2 [file Table2.DOCX]

**Table S2| Major viral families in the Lake Ontario and Lake Erie viromes (VLP Fractions)**

| **Virus Family** | **Host** | **Relative Abundance (% of viral reads)** | | | | | | |
| --- | --- | --- | --- | --- | --- | --- | --- | --- |
|  |  | **Lake Ontario** | | | | **Lake Erie** | | |
|  |  | **Lakeside Beach** | **Fifty Point Beach** | **Queen’s Royal Beach** | **Long Beach** | | **Long Beach Conservation Area East** | **Nickel Beach** |
| *Myoviridae* | Bacteria | 80.76 | 82.10 | 81.18 | 79.66 | | 79.33 | 80.98 |
| *Podoviridae* | Bacteria | 4.74 | 4.87 | 4.77 | 8.81 | | 7.99 | 6.31 |
| *Siphoviridae* | Bacteria | 4.41 | 4.63 | 4.73 | 3.83 | | 3.82 | 4.37 |
| Unclassified (*Caudovirales* order) | Bacteria | 0.78 | 0.86 | 0.96 | 0.88 | | 1.10 | 0.73 |
| *Microviridae* | Bacteria | - | - | 0.01 | - | | - | - |
| *Inoviridae* | Bacteria | - | - | - | 0.01 | | - | - |
| *Phycodnaviridae* | Algae | 6.12 | 4.51 | 5.25 | 4.12 | | 4.04 | 3.61 |
| *Iridoviridae* | Insects, Amphibians, Fish, Invertebrates | 2.97 | 2.85 | 2.95 | 2.57 | | 3.54 | 3.82 |
| *Poxviridae* | Humans and other vertebrates, Arthropods | 0.05 | 0.07 | 0.04 | 0.04 | | 0.09 | 0.09 |
| *Alloherpesviridae* | Fish, Amphibians | 0.02 | 0.02 | - | 0.03 | | 0.03 | 0.04 |
| *Herpesviridae* | Animals including humans | 0.05 | 0.04 | 0.01 | 0.03 | | - | 0.03 |
| *Marseilleviridae* | Amoeba | 0.06 | 0.01 | - | 0.01 | | - | - |
| *Baculoviridae* | Insects | 0.03 | 0.04 | 0.08 | 0.02 | | 0.03 | 0.01 |
| *Adenoviridae* | Humans and other vertebrates | 0.02 | - | - | - | | - | - |
| *Nimaviridae* | Crustaceans | 0.02 | - | 0.01 | - | | - | - |
