# Supplementary material for: Spatial and temporal dynamics of virus occurrence in two freshwater lakes captured through metagenomic analysis
Source: Front Microbiol. 2015 Sep 15;6:960. doi: 10.3389/fmicb.2015.00960 (PMC4569853; doi:10.3389/fmicb.2015.00960)
Supplement: Supplementary file 3 [file Table3.DOCX]

**Table S3| Variation in relative abundance of virus families from Lakeside and Long Beach**

| **Virus Family** | **% of total viral reads mapped to top five Viral Families** | | | | | | | | | |
| --- | --- | --- | --- | --- | --- | --- | --- | --- | --- | --- |
|  | **Lakeside Beach (VLP Fraction)** | | | | | **Long Beach (VLP Fraction)** | | | | |
|  | **Lakeside Beach - 1** | **Lakeside Beach - 2** | **Lakeside Beach - 3** | **Mean** | **Coefficient of Variation** | **Long Beach - 1** | **Long Beach - 2** | **Long Beach - 3** | **Mean** | **Coefficient of Variation** |
| Myoviridae | 80.76 | 82.74 | 81.84 | 81.78 | 1.21 | 79.66 | 82.69 | 48.67 | 70.34 | 26.77 |
| Podoviridae | 4.74 | 5.12 | 6.67 | 5.51 | 18.60 | 8.81 | 6.47 | 22.16 | 12.48 | 67.81 |
| Siphoviridae | 4.41 | 4.05 | 4.03 | 4.16 | 5.16 | 3.83 | 3.46 | 12.34 | 6.54 | 76.81 |
| Phycodnaviridae | 6.12 | 3.46 | 3.79 | 4.46 | 32.49 | 4.12 | 3.75 | 10.24 | 6.03 | 60.41 |
| Iridoviridae | 2.97 | 3.41 | 2.85 | 3.08 | 9.71 | 2.57 | 2.74 | 1.96 | 2.42 | 16.83 |
