# Supplementary material for: Spatial and temporal dynamics of virus occurrence in two freshwater lakes captured through metagenomic analysis
Source: Front Microbiol. 2015 Sep 15;6:960. doi: 10.3389/fmicb.2015.00960 (PMC4569853; doi:10.3389/fmicb.2015.00960)
Supplement: Supplementary file 6 [file Image1.PDF]

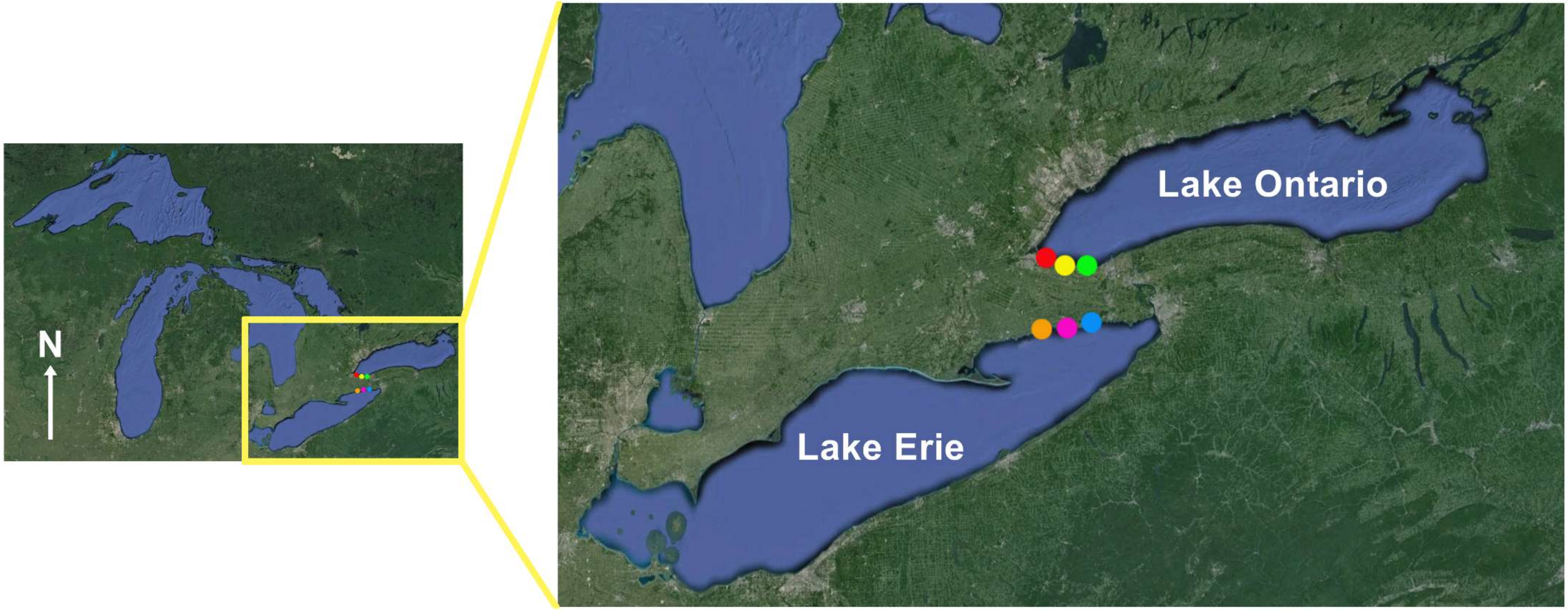

Figure S1. Sampling Sites within the Great Lakes. Samples were collected from six different beaches of Lake Ontario and Lake Erie. The Lake Ontario beaches (Fifty Point, Lakeside and Queen' Royal Beaches) are labeled as red, yellow and green dots respectively while the orange, pink and blue dots represent the Lake Erie beaches (Long Beach, Long Beach Conservation Area East and Nickel Beach respectively). Satellite Image: Google Maps.
